# Wnt signaling regulates hepatocyte cell division by a transcriptional repressor cascade

**DOI:** 10.1073/pnas.2203849119

**Published:** 2022-07-22

**Authors:** Yinhua Jin, Teni Anbarchian, Peng Wu, Abby Sarkar, Matt Fish, Weng Chuan Peng, Roel Nusse

**Affiliations:** ^a^Department of Developmental Biology, Institute for Stem Cell Biology and Regenerative Medicine, Stanford University School of Medicine, Stanford, CA 94305;; ^b^HHMI, Stanford University School of Medicine, Stanford, CA 94305;; ^c^Department of Pediatrics, Stanford University School of Medicine, Stanford, CA 94305

**Keywords:** liver, Wnt, proliferation

## Abstract

Cell proliferation is tightly controlled by inhibitors that block cell cycle progression until growth signals relieve this inhibition, allowing cells to divide. In several tissues, including the liver, cell proliferation is inhibited at mitosis by the transcriptional repressors E2F7 and E2F8, leading to formation of polyploid cells. Whether growth factors promote mitosis and cell cycle progression by relieving the E2F7/E2F8-mediated inhibition is unknown. We report here on a mechanism of cell division control in the postnatal liver, in which Wnt/β-catenin signaling maintains active hepatocyte cell division through *Tbx3*, a Wnt target gene. The TBX3 protein directly represses transcription of *E2f7* and *E2f8*, thereby promoting mitosis. This cascade of sequential transcriptional repressors, initiated by Wnt signals, provides a paradigm for exploring how commonly active developmental signals impact cell cycle completion.

## Significance

As a general model for cell cycle control, repressors keep cells quiescent until growth signals remove the inhibition. For S phase, this is exemplified by the Retinoblastoma (RB) protein and its inactivation. It was unknown whether similar mechanisms operate in the M phase. The Wnt signaling pathway is an important regulator of cell proliferation. Here, we find that Wnt induces expression of the transcription factor *Tbx3*, which in turn represses mitotic inhibitors *E2f7* and *E2f8* to permit mitotic progression. Such a cascade of transcriptional repressors may be a general mechanism for cell division control. These findings have implications for tissue homeostasis and disease, as the function for Wnt signaling in mitosis is relevant to its widespread role in stem cells and cancer.

Cells in adult tissues remain mostly quiescent unless they become activated by growth factors to enter the cell cycle and proliferate (1). Quiescence is commonly imposed by inhibitors that prevent cells from entering S phase (2). To allow cell cycle entry, growth factors repress these inhibitors and activate the G1/S transition (3). In some tissues, including the liver, cells can enter S phase to duplicate their genome, but do not complete M phase, thus becoming polyploid (4–6). In the liver, two members of the E2F family of cell cycle regulators, *E2f7* and *E2f8*, are responsible for this form of cell cycle arrest (7, 8). Expression of *E2f7* and *E2f8* is induced at the time of weaning when the tissue switches from initially containing diploid hepatocytes to becoming mostly polyploid (4, 9, 10). Acting as mutually redundant transcriptional repressors, E2F7 and E2F8 inhibit the expression of many genes, including *Aurka/b*, *Ccnb1*, and *Plk1*, which encode proteins that act during mitotic progression (7, 8, 11). Deleting *E2f7* and *E2f8* from hepatocytes results in completion of M phase and the generation of diploid daughter cells (7, 8). Therefore, in the liver, DNA synthesis is uncoupled from cell proliferation due to the activity of E2F7 and E2F8. Whether growth factors can suppress the activity of *E2f7* and *E2f8* and thereby promote cell division is currently not known.

Hepatocytes in the postnatal liver expand rapidly in a Wnt/β-catenin signaling-dependent way (12–16). In the mouse and human liver, Wnt ligands are produced by endothelial cells of the central vein branches and the nearby sinusoids (17–19), creating a zonated expression pattern of Wnt target genes and key metabolic enzymes in pericentral hepatocytes (20–22). Among the genes highly expressed in the pericentral zone is the transcriptional repressor *Tbx3* (17, 19). *Tbx3* is required for embryonic liver development and promotes hepatic progenitor proliferation likely by repressing the *p19^ARF^(Cdkn2a)* cell cycle inhibitor (23). *Tbx3* also maintains expression of hepatocyte-lineage genes, such as *Cebpα* and *Hnf4α*, and represses the cholangiocyte fate (23, 24). In hepatic tumor cells with activating *β-catenin* mutations, *Tbx3* is overexpressed and mediates cell proliferation and survival (25). These findings highlight the roles of *Tbx3* in hepatic cell cycle control during liver development and tumorigenesis.

In the work presented here, we have studied the regulation of hepatocyte cell division in vivo during postnatal liver growth as well as in primary cell culture. We report that Wnt growth factors induce *Tbx3* expression, which subsequently represses *E2f7* and *E2f8* to regulate cell division. We conclude that Wnt signals regulate the cell cycle at the level of mitosis through this cascade of repressive interactions.

## Results

### *Tbx3* Promotes Hepatocyte Proliferation Downstream of Wnt Signaling.

**Fig. 1.**
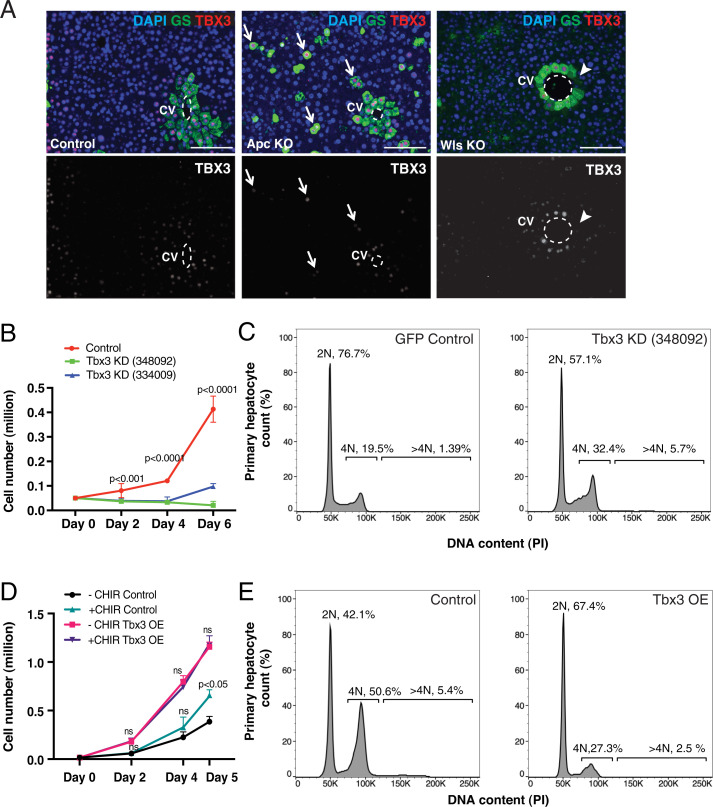
*Tbx3* acts downstream of Wnt signaling and promotes proliferation of cultured primary hepatocytes. (*A*) *Tbx3* expression is downstream of Wnt signaling. Immunofluorescence for GS and TBX3 is shown in control livers, CRISPR-Cas9–generated Apc KO cells and Wls KO (*Ve-CadCreERT2; Wls^f/f^*) livers. Arrows: Apc KO cells that express *Tbx3*. Arrowheads: Wls KO cells that do not express *Tbx3*. Dashed lines delineate central veins. (*B*) *Tbx3* knockdown (Tbx3 KD) slows down hepatocyte proliferation. Growth curves show the number of cells in control (GFP) and two different Tbx3 KD (shRNA #348092 and #334009) mouse primary hepatocyte cultures at days 0, 2, 4, and 6 after seeding. *P* values represent comparisons between control and each Tbx3 KD condition from three independent experiments. (*C*) *Tbx3* knockdown leads to increased numbers of polyploid hepatocytes in culture. Representative flow cytometry plots show ploidy distribution of control and Tbx3 KD cells, stained with PI. Percentages of ploidy classes are reported as averages from three independent experiments. (*D*) *Tbx3* overexpression (OE) is sufficient to increase hepatocyte proliferation, in the absence of activated Wnt signaling. Growth curves of control (*EF1α-GFP*) or *Tbx3* overexpressing (Tbx3 OE, *EF1α-GFP-P2A-Tbx3*) primary hepatocytes with or without added Wnt-activator CHIR99021 (CHIR) are shown at days 0, 2, 4, and 5 after seeding. Statistical comparisons were made within control groups and Tbx3 OE groups. (*E*) *Tbx3* overexpression in primary hepatocytes leads to a higher percentage of diploid cells. Representative flow cytometry plots for ploidy distribution of control and Tbx3 OE hepatocytes, stained with PI. Percentages of ploidy distribution are averages from three independent experiments. Statistical significance was determined by Student’s *t* test. Error bars represent SDs from three independent replicates. (Scale bars, 100 µm.) CV, central vein; ns, not significant.

Similar to the adult liver, *Tbx3* is expressed in Wnt-responsive pericentral hepatocytes during postnatal liver growth (*SI Appendix*, Fig. S1*A*). To determine whether *Tbx3* is a target of Wnt signaling in the liver, we either overactivated or eliminated the Wnt pathway in the tissue, and used Glutamine synthetase (GS), a Wnt target (26), to assess pathway activity. Using CRISPR-Cas9 gene editing, we deleted *Apc*, a negative regulator of Wnt signal transduction in hepatocytes, and observed ectopic expression of *Tbx3* (Fig. 1*A*). Conversely, mice carrying mutations for the Wnt secretion machinery gene *Wntless* *(Wls)* exhibited a partial loss of *Tbx3* expression (Fig. 1*A*). These findings agree with *Tbx3* acting as a target of Wnts in other contexts (25, 27–30).

To examine the function of *Tbx3* in hepatocytes under defined conditions, we employed a recently developed method for long-term culture and genetic manipulation of primary hepatocytes, where cells multiply rapidly in a Wnt-dependent manner (31). We knocked down *Tbx3* in cultured hepatocytes using two different shRNA constructs (*SI Appendix*, Fig. S1*B*). This resulted in significant slowing down of hepatocyte proliferation (Fig. 1*B*) and increased numbers of polyploid cells (Fig. 1*C* and *SI Appendix*, Fig. S1*C* and Table S1), suggesting that hepatocytes failed to complete M phase. Conversely, overexpression of *Tbx3* in hepatocytes was sufficient to increase proliferation (Fig. 1*D* and *SI Appendix*, Fig. S1*D* and *E*), even in the absence of CHIR99021, a GSK3 inhibitor that activates Wnt signaling (Fig. 1*D*). Additionally, overexpression of *Tbx3* increased the percentage of diploid cells (Fig. 1*E* and *SI Appendix*, Table S1). As expected, CHIR99021 alone enhanced proliferation of hepatocytes albeit not to the same degree as *Tbx3* overexpression (Fig. 1*D*).

### Deletion of *Tbx3* and Changes in Ploidy.

**Fig. 2.**
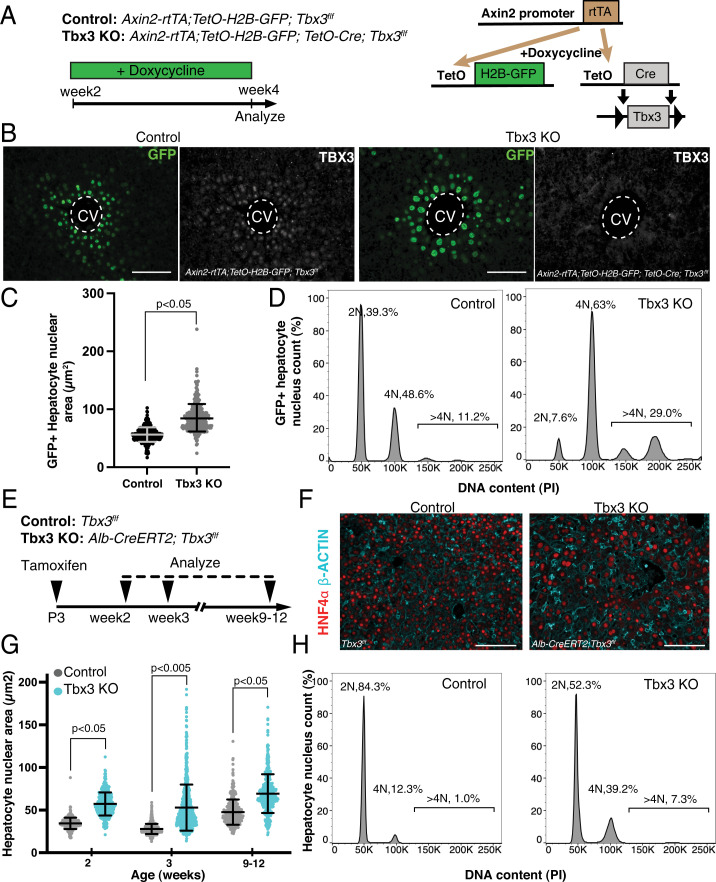
*Tbx3* ablation induces polyploidization of hepatocytes. (*A*) Schematic representation of mouse genotypes and experimental timeline. Animals were placed on a regimen of doxycycline-treated drinking water from postnatal week 2 to week 4, to induce Axin2-rtTA–driven expression of GFP and deletion of *Tbx3* in pericentral hepatocytes (when the TetO-Cre transgene is present). (*B*) Axin2-rtTA–driven GFP expression and *Tbx3* deletion. Representative images of pericentral GFP^+^ nuclei detected by immunofluorescence, at postnatal week 4 in control (*Axin2-rtTA; TetO-H2B-GFP; Tbx3^f/f^*) and Tbx3 KO (*Axin2-rtTA; TetO-H2B-GFP; TetO-Cre; Tbx3^f/f^*) livers are shown. TBX3 immunofluorescence shows efficient deletion of the protein in Tbx3 KO livers. (*C*) Axin2-rtTA–driven loss of *Tbx3* results in increased hepatocyte nuclear area. Distribution of GFP^+^ nuclear area at postnatal week 4 is shown. Bars indicate mean and SD of all measured nuclei. *t* test comparison with Welch’s correction was performed on the mean nuclear area of *n* = 3 mice. (*D*) Axin2-rtTA–driven loss of *Tbx3* results in increased hepatocyte nuclear ploidy. Nuclear ploidy distribution of GFP^+^ hepatocytes in control and Tbx3 KO mice, stained with PI and measured by flow cytometry at postnatal week 4 is shown. Plots shown are chosen as representatives of each genotype from *n* = 3 animals and noted percentages are averages. (*E*) Schematic representation of Alb-CreERT2–driven *Tbx3* deletion. Tamoxifen was administered to P3 neonates with control (*Tbx3^f/f^*) or Tbx3 KO (*Alb-CreERT2; Tbx3^f/f^*) genotypes. Livers were harvested at postnatal weeks 2, 3, and 9 to 12 for analysis. (*F*) Visualization of hepatocyte membranes and nuclei in Alb-CreERT2–driven Tbx3 KO livers. Representative HNF4α and β-ACTIN immunofluorescence images from control and Tbx3 KO livers at postnatal week 2 are shown. (*G*) Alb-CreERT2–driven loss of *Tbx3* results in increased hepatocyte nuclear area. Distribution of HNF4α^+^ nuclear area is shown. Bars indicate mean and SD for all measured nuclei per genotype. *t* test comparison with Welch’s correction was performed on the mean nuclear area from *n* = 3 animals per genotype and timepoint. (*H*) Alb-CreERT2–driven loss of *Tbx3* results in increased hepatocyte nuclear ploidy. Nuclear ploidy distribution of hepatocytes stained with PI and measured by flow cytometry at postnatal week 2. (Scale bars, 100 µm.) Dashed lines delineate CVs.

We examined *Tbx3* function in the growing postnatal liver by generating inducible *Tbx3* loss-of-function mutant mice (Tbx3 knockout [KO]) carrying the *Axin2-rtTA; TetO-H2B-GFP; TetO-Cre; Tbx3^f/f^* genotype. Axin2-rtTA is a doxycycline-inducible transgene, which leaves the endogenous *Axin2* gene intact and is expressed in a pattern similar to *Tbx3* (Fig. 2*A*) (17). To obtain maximal elimination of *Tbx3*, doxycycline was administered from postnatal week 2 to week 4, at which point tissues were harvested for analysis (Fig. 2*B*). Nuclear size measurement (Fig. 2*C*) and nuclear DNA content analysis (Fig. 2*D*) of GFP-labeled hepatocytes revealed a significant increase in the proportion of polyploid nuclei in Tbx3 KO livers (>2N: 59.8% in control and 92% in Tbx3 KO) (*SI Appendix*, Table S1). The decrease in diploidy and the concomitant increase in polyploidy indicated that in the absence of *Tbx3*, hepatocytes were able to complete S phase but not M phase. In parallel, we used the inducible and hepatocyte-specific Albumin-CreERT2 (Alb-CreERT2) driver to eliminate *Tbx3* in all hepatocytes. We administered a single dose of tamoxifen at postnatal day 3 (P3) and analyzed the livers at different timepoints into adulthood (Fig. 2*E* and *SI Appendix*, Fig. S2*A* and *B*). Loss of *Tbx3* did not affect liver shape or relative mass, at any timepoint (*SI Appendix*, Fig. S2*C* and *D*). Consistent with the observations above, Tbx3 KO livers were comprised of larger nuclei at all analyzed timepoints compared to control (Fig. 2*F* and *G*) and DNA content analysis confirmed increased ploidy in mutant tissues (Fig. 2*H* and *SI Appendix*, Table S1). Taken together, we conclude that *Tbx3* regulates polyploidization of hepatocytes in vivo and in vitro by its control over cell division.

### TBX3 Represses Transcription of *E2f7* and *E2f8*.

**Fig. 3.**
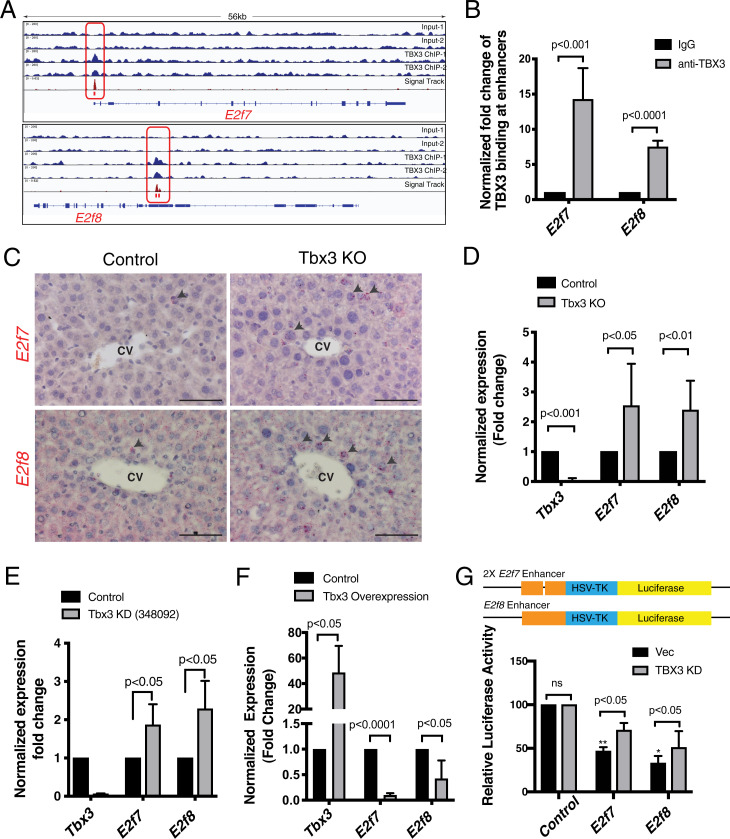
TBX3 directly represses transcription of *E2f7* and *E2f8*. (*A*) TBX3 binds to *E2f7* promoter and *E2f8* enhancer, shown by ChIP-Seq binding profiles (reads per million per base pair) for TBX3 at the *E2f7* and *E2f8* loci in primary hepatocytes. Red boxes indicate significant binding peaks. These sites are tested in *B* and *G*. Profiles are representative of two independent biological replicates. (*B*) Validation of binding sites outlined in *A* by ChIP-qPCR. (*C*–*E*) *E2f7* and *E2f8* transcripts are ectopically up-regulated upon deletion of *Tbx3*. (*C*) mRNA in situ hybridization for *E2f7* and *E2f8* in control (*Axin2-rtTA; TetO-H2B-GFP; Tbx3^f/f^*) and Tbx3 KO (*Axin2-rtTA; TetO-H2B-GFP; TetO-Cre; Tbx3^f/f^*) livers at postnatal week 4 show increased signal in the pericentral hepatocytes of Tbx3 KO livers. Arrowheads point to positive signals. (*D*) qRT-PCR analysis of *Tbx3*, *E2f7*, and *E2f8* transcripts from whole livers of the same samples as in *C* show significantly increased levels of *E2f7* and *E2f8* in the absence of *Tbx3*. (*E*) qRT-PCR analysis shows increased *E2f7* and *E2f8* transcription in Tbx3 KD cultured mouse primary hepatocytes. (*F*) *E2f7* and *E2f8* are down-regulated upon overexpression of *Tbx3*. qRT-PCR analysis of *Tbx3*, *E2f7*, and *E2f8* transcripts in control (*EF1α-GFP*) and Tbx3 OE (*EF1α-GFP-P2A-Tbx3*) mouse primary hepatocytes. (*G*) TBX3 represses *E2f7* and *E2f8* expression by binding to their regulatory regions. Regions outlined in *A* as TBX3 binding sites were cloned into luciferase expression constructs for functional analyses in HepG2 cells and luciferase activity was measured in vector control or TBX3 KD cells. *P* values noted via asterisks are comparisons of control vs. *E2f7* and control vs. *E2f8* in the vector control condition. *P < 0.05; **P < 0.01. Statistical significance was determined by Student’s *t* test from *n* = 3 independent experiments. Error bars indicate SD. (Scale bars, 100 µm.) ns, not significant.

To identify mechanisms of *Tbx3* function, a known repressor of transcription, we investigated potential target genes of TBX3 using chromatin immunoprecipitation-sequencing (ChIP-Seq) in *Tbx3*-overexpressing mouse primary hepatocytes. We found that the TBX3 protein binds to promoter and enhancer regions of *E2f7* and *E2f8*, respectively, and verified this interaction by ChIP-qPCR (Fig. 3*A* and *B*). This suggested that TBX3 represses *E2f7* and *E2f8*, and indeed *E2f7* and *E2f8* transcripts were both ectopically increased in Tbx3 KO livers (Fig. 3*C* and *D*). Knockdown and overexpression of *Tbx3* in cultured primary hepatocytes further corroborated the interactions with *E2f7* and *E2f8* (Fig. 3*E* and *F*).

To verify that repression of *E2f7* and *E2f8* by TBX3 occurs on the regulatory sequences of these genes, we employed a luciferase reporter assay in human hepatoblastoma HepG2 cells, which express *TBX3* at high levels (32). Addition of the mouse *E2f7* or *E2f8* enhancer regions containing the TBX3 binding sites to an HSV-TK reporter construct led to significant repression of the luciferase reporter activity compared to control vector with HSV-TK promoter only (Fig. 3*G*). Moreover, reporter activity was increased when *TBX3* was knocked down or T-box binding motifs were mutated or deleted from the enhancer regions, confirming that TBX3 is a specific transcriptional repressor of *E2f7* and *E2f8* (Fig. 3*G* and *SI Appendix*, Fig. S3*A*). In the adult human liver, *TBX3* is specifically expressed in pericentral hepatocytes, similar to the mouse liver (*SI Appendix*, Fig. S3*B*). Analysis of ChIP-Seq data from the ENCODE database (GSE105374) (33, 34) showed that human TBX3 protein also binds to *E2F7* and *E2F8* enhancers (*SI Appendix*, Fig. S3*C*), suggesting that TBX3 has conserved functions in both human and mouse livers. Hence, a variety of different experiments provided evidence that TBX3 directly regulates *E2F7* and *E2F8* expression.

### Epistatic Relationships between *Tbx3* and *E2f7/E2f8*.

**Fig. 4.**
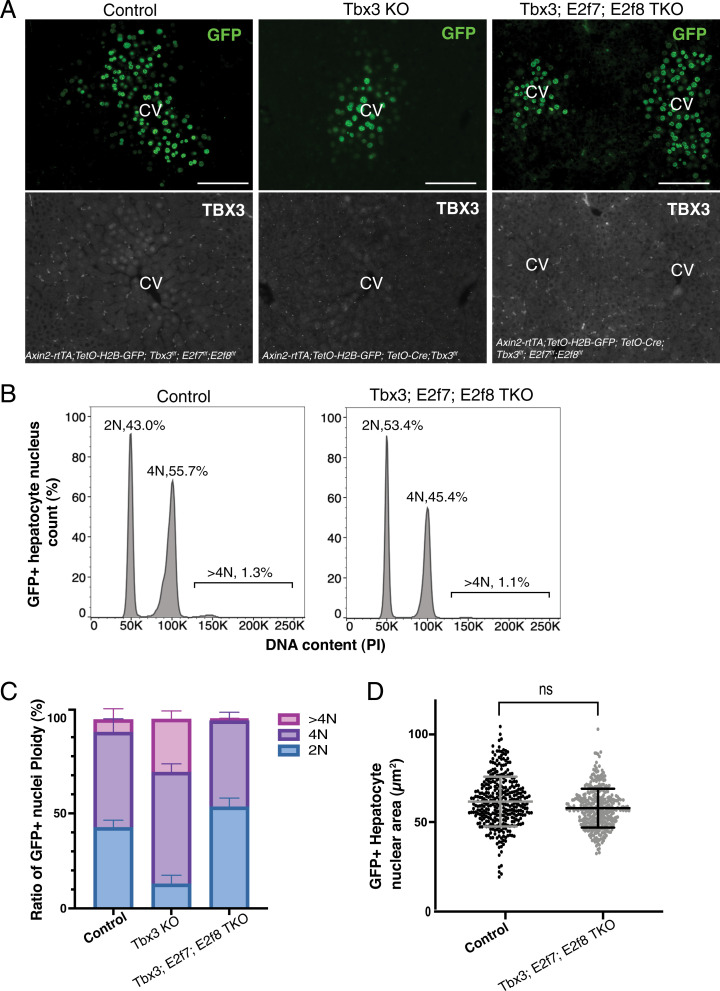
Genetic epistasis test showing *Tbx3* controls hepatocyte ploidy by repressing *E2f7* and *E2f8*. (*A*) Visualization of Axin2-rtTA–driven GFP expression in control, Tbx3 KO, or Tbx3*-*E2f7*-*E2f8 TKO livers. Representative images of GFP and TBX3 immunofluorescence in the pericentral zone in control (*Axin2-rtTA; TetO-H2B-GFP; Tbx3^f/f^; E2f7^f/f^; E2f8^f/f^*), Tbx3 KO (*Axin2-rtTA; TetO-H2B-GFP; TetO-Cre; Tbx3^f/f^*), and Tbx3*;* E2f7*;* E2f8 TKO (*Axin2-rtTA; TetO-H2B-GFP; TetO-Cre; Tbx3^f/f^; E2f7^f/f^; E2f8^f/f^*) livers at postnatal week 4 are shown. (*B*–*D*) Deletion of *E2f7* and *E2f8* along with *Tbx3* restores the balance of ploidy. (*B*) Nuclear ploidy plots of GFP^+^ hepatocytes in control and Tbx3-E2f7-E2f8 TKO mice, stained with PI and measured by flow cytometry. Plots shown are chosen as representatives of each genotype and noted percentages are averages from *n* = 3 control and *n* = 4 Tbx3-E2f7-E2f8 TKO mice. (*C*) Bar graph summarizes nuclear ploidy distribution of GFP^+^ nuclei from each genotype at postnatal week 4. (*D*) Measurements of nuclear area from GFP^+^ pericentral hepatocytes in control (*n* = 3 mice) and Tbx3-E2f7-E2f8 TKO (*n* = 4 mice). Bars indicate mean and SD. *P* = 0.2249, *t* test with Welch’s correction comparison of mean nuclear area from biological replicates. (Scale bars, 100 µm). ns, not significant.

The negative interactions between *Tbx3* and *E2f7*/*E2f8* and the up-regulation of *E2f7* and *E2f8* in the absence of *Tbx3* would imply that loss of all three genes would suppress the *Tbx3* knockout phenotype. To test possible epistatic interactions by mouse genetics, we generated triple conditional mutants of *Tbx3*, *E2f7*, and *E2f8* (Tbx3*-*E2f7*-*E2f8 TKO). First, we used the *Axin2-rtTA; TetO-GFP; TetO-Cre* system to delete all three genes by continuously administering doxycycline from postnatal week 2 to week 4 (Fig. 4*A*). GFP-labeled nuclei in Tbx3*-*E2f7*-*E2f8 TKO livers were indistinguishable from controls in ploidy and size (Fig. 4*B*–*D* and *SI Appendix*, Table S1). Similar results were obtained in the pan-hepatocyte deletion model of Tbx3*-*E2f7*-*E2f8 TKO livers (*SI Appendix*, Fig. S4*A*–*C* and Table S1). These findings show that simultaneous removal of *E2f7*, *E2f8*, and *Tbx3* resolves the polyploid phenotype caused by removal of *Tbx3* alone. These genetic interactions imply a linear pathway whereby Wnt activates *Tbx3*, which represses the mitotic inhibitors *E2f7* and *E2f8*; this therefore relieves cell cycle arrest and maintains hepatocytes in a diploid, dividing state.

### Loss of *Tbx3* Leads to Fibrosis.

**Fig. 5.**
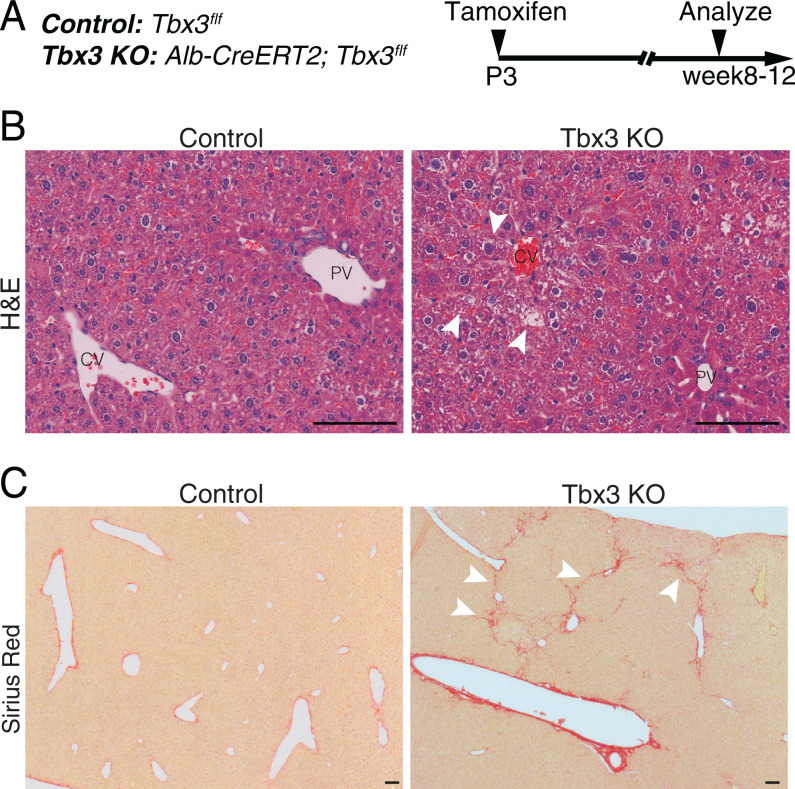
Physiological impact of long-term *Tbx3* deletion. (*A*) Experimental timeline. Control (*Tbx3^f/f^*) or Tbx3 KO (*Alb-CreERT2; Tbx3^f/f^*) neonates were administered tamoxifen at P3 and liver sections were analyzed in adults (8 to 12 wk old). (*B*) Representative images of hematoxylin and eosin (H&E) sections from adult livers are shown. Arrowheads point to cytoplasmic anomalies in the pericentral zone. (*C*) Representative images of Sirius Red–stained sections from adult livers are shown. Arrowheads point to fibrotic tracts that were apparent in five of eight Tbx3 KO mice and none of the five control mice. (Scale bars, 100 µm). PV, portal vein.

Despite the functions attributed here to *Tbx3* in postnatal liver growth, we did not observe major changes in liver weight or morphology (*SI Appendix*, Fig. S2*C* and *D*). To identify physiological impact of long-term loss of *Tbx3* on the liver, we ablated the gene in neonates with the Alb-CreERT2 driver and aged the animals to adulthood (Fig. 5*A*). Upon histological analysis, we observed cytoplasmic vacuoles and anomalies in the pericentral zone (Fig. 5*B*). In addition, collagen staining revealed fibrotic areas in five of eight adult Tbx3 KO livers, while none of the five control animals exhibited fibrosis (Fig. 5*C*).

## Discussion

In contrast to the extensive insight on the role of growth factors that initiate S phase of the cell cycle, little is known about extracellular signals that regulate M phase. The Wnt/β-catenin signaling pathway is one of the mechanisms known to control G1/S transition (35–38). A role for Wnt signaling during M phase has also been suggested based on high Wnt/β-catenin signaling activity during cell division, through an unknown mechanism (39, 40). In this work we have shown that in the liver, M phase is regulated by Wnt signals through the Wnt target transcription factor *Tbx3*, which in turn represses the mitotic inhibitors *E2f7* and *E2f8*. Interestingly, activating mutations in Wnt signaling components are common in liver cancer (41, 42), while *E2f7* and *E2f8*, as well as polyploidy, are known to suppress tumorigenesis (43–46). These mitotic inhibitors are expressed in several other tissues, such as the placenta and pancreas, where they are also implicated in arresting the cell cycle (8, 47–49). Whether Wnt signaling likewise regulates mitosis by suppressing *E2f7* and *E2f8* in other tissues and contexts is relevant to identifying the widespread functions of Wnts in development and disease (41).

Our data indicated that *Tbx3*, acting in the Wnt signaling network, fine tunes the degree and onset of zonated polyploidy in the liver, by promoting mitosis and cell division. *Tbx3*, which has been shown to act in a dosage-sensitive manner in other contexts (50), seems to be expressed in a gradient in the pericentral zone (*SI Appendix*, Fig. S1*A*). Whether there are differences in *Tbx3* function or rate of proliferation in TBX3-high cells adjacent to the central vein and TBX3-low cells further into the lobule is unclear. *Tbx3*-expressing cells may proliferate at different rates or capacities in the context of injury when additional cell cycle inducers promote tissue repair (19). Moreover, *Tbx3*-negative hepatocytes in the periportal zone seem to maintain low ploidy levels through the course of the animal’s lifetime (51). Whether mitosis is regulated by a periportal signal in these cells remains to be studied.

While we did not observe major changes in liver weight or morphology due to loss of *Tbx3* or increased polyploidy, the fibrotic phenotype of Tbx3 KO mice indicates an important role for cell cycle regulation in the tissue. In the absence of a capacity for cell division, fibrosis becomes a mode of tissue repair and leads to tissue scarring (52, 53). Appearance of fibrotic areas in Tbx3 KO livers suggests that loss of *Tbx3* is not compatible with long-term homeostatic tissue renewal, likely due to failure in cell division, which leads to compensatory fibrotic repair.

Regarding an absence of growth abnormalities during postnatal development, we hypothesize that the increased polyploidy itself contributes to growth of the tissue and compensates for the lack of cell division, as it has been observed with partial hepatectomy (54). In addition, in the growing and adult liver, *Tbx3* is expressed primarily in the pericentral zone, and it is likely that cells from other zones are able to compensate and fill the growth gap in the absence of *Tbx3*.

Our explorations of the mammalian liver highlight it as a useful and unique model for cell cycle studies. Hepatocytes have distinct modes of cell cycle activity. They first undergo complete cycles and proliferate, then face roadblocks and become polyploid. The temporal regulation of these events provides distinct windows to query the extracellular cues that drive the cell cycle and to understand how these signals are linked to the intrinsic cell cycle machinery.

## Materials and Methods

### Animals.

The Institutional Animal Care and Use Committee at Stanford University approved all animal methods and experiments. Wild-type C57BL/6J mice, *Axin2-rtTA* [B6.Cg-Tg(Axin2-rtTA2S*M2)7Cos/J] (55)*, TetO-H2B-GFP* [*Tg(tetO-HIST1H2BJ/GFP)47Efu/J*] (56), and *TetO-Cre* [*B6.Cg-Tg(tetO-cre)1Jaw/J*], *Wls^f/f^* (*129S-Wls^tm1^0.1Lan/J*) (57) strains were obtained from The Jackson Laboratory (JAX). *Tbx3^f/f^* mice were a gift from Anne Moon (Geisinger Clinic, Danville, PA) (58). *Alb-CreERT2* mice were a gift from Julien Sage (Stanford University, Stanford, CA) (59). *E2f7^f/+^; E2f8^f/+^* were gifted by Alain de Bruin (Utrecht University, Utrecht, The Netherlands) (60) and were rederived at the Stanford Transgenic Facility. *Cdh5-CreERT2* mice were used as previously described (61). For knocking out *Tbx3* alone or *Tbx3; E2f7; E2f8* together using the Axin2-rtTA driver, animals were given 1 mg/mL doxycycline (Sigma D9891) in drinking water from P14 until P28. In experiments involving the Alb-CreERT2 driver, neonatal P3 mice received a single intragastric injection of 0.08 mg tamoxifen (Sigma T5648), dissolved in corn oil with 10% ethanol. For knocking out *Wls*, *Cdh5-CreERT2; Wls^f/f^* mice 8 to 10 wk of age received intraperitoneal injections of tamoxifen on 4 consecutive days and tissues were harvested at 7 d after the last dose of tamoxifen. All mice were housed with a 12-h light/dark cycle with ad libitum access to water and normal chow.

### CRISPR-Cas9–Mediated *Apc* Deletion.

The adeno-associated virus with a single guide RNA targetting the *Apc* gene (AAV-sgApc) was produced from the pAAV-Guide-it-Down construct (Clontech Laboratories Inc., 041315) using assembly primers: (forward) 5’CCGGAGGCTGCATGAGAGCACTTG3’ and (reverse) 5’AAACCAAGTGCTCTCATGCAGCCT3’3. AAV-sgApc contains a U6 promoter and an sgRNA targeting the sequence 5’AGGCTGCATGAGAGCACTTG3’ in exon 13 of *Apc*. Adult CRISPR-Cas9 knockin mice were obtained from JAX and a single intraperitoneal injection of AAV-sgApc was administered at a dose of 1 × 10^13 genome copies/kg. Livers were collected for analysis 4 wk after induction.

### Tissue Collection and Processing.

Livers were collected, fixed overnight at room temperature in 10% neutral buffered formalin, dehydrated, cleared in HistoClear (Natural Diagnostics), and embedded in paraffin. Sections were cut at 5-μm thickness, deparaffinized, rehydrated, and processed for further staining via immunofluorescence or in situ hybridization assays as described below.

### Immunofluorescence and Immunocytochemistry.

Tissue slides were subjected to antigen retrieval with Tris buffer pH = 8.0 (Vector Labs H-3301) in a pressure cooker. They were then blocked in 5% normal donkey serum in phosphate buffered saline (PBS) containing 0.1% Triton-X, in combination with the avidin/biotin blocking reagent (Vector Labs SP-2001). Sections were incubated with primary and secondary antibodies and mounted in Prolong Gold with DAPI medium (Invitrogen). Biotinylated goat antibody (Jackson Immunoresearch 705-065-147) was applied to sections stained with TBX3, before detection with Streptavidin-647. GS and β-ACTIN staining was performed with the Mouse-on-Mouse detection kit (Vector Labs) according to the manufacturer’s protocol. The following antibodies were used: GFP (chicken, 1:500; Abcam ab13970), TBX3 (goat, 1:50; Santa Cruz sc-17871), GS (mouse, 1:500; Millipore MAB302), β-ACTIN (mouse, 1:100; Abcam ab8226), and HNF4α (rabbit 1:50; Santa Cruz sc8987). Samples were imaged at 20× magnification using a Zeiss Imager Z.2 and processed and analyzed with ImageJ software. For immunocytochemistry, plated cells were fixed with 4% paraformaldehyde, blocked in 5% normal donkey serum in PBS containing 0.1% Triton-X, and stained with primary and secondary antibodies as indicated above. Cells were imaged using a Zeiss Spinning Disk Confocal Microscope.

### mRNA In Situ Hybridization.

In situ hybridizations were performed using the manual RNAscope 2.5 HD Assay-Red Kits (Advanced Cell Diagnostics) according to the manufacturer’s instructions. Images were taken at 20× magnification on a Zeiss Imager Z.2 and processed using ImageJ software. Probes used in this study were *E2f7* (target region: 612 to 1,526) and *E2f8* (target region: 911 to 1,893).

### RNA Isolation and qRT-PCR.

Liver samples were homogenized in TRIzol (Invitrogen) with Pestle Motor Mixer (Argos Technologies A0001) or bead homogenizer (Sigma). Total RNA was purified using the RNeasy Mini Isolation Kit (Qiagen) and reverse transcribed (High-Capacity cDNA Reverse Transcription Kit; Life Technologies) according to the manufacturer’s protocol. qRT-PCRs were performed with TaqMan Gene Expression Assays (Applied Biosystems) on a StepOnePlus Real-Time PCR System (Applied Biosystems). Relative target gene expression levels were calculated using the delta-delta CT method as previously described (62). Gene expression assays used were *Gapdh* (Mm99999915_g1) as control, *Tbx3* (Mm01195719_m1), *E2f7* (Mm00618098_m1), and *E2f8* (Mm01204165_g1).

### Hepatocyte Isolation and Culture.

Hepatocytes were isolated by a two-step collagenase perfusion technique as previously described (31). Six-well plates (Greiner Bio-One 657160) were precoated with collagen (Corning 354236). Primary hepatocytes were plated into 2 mL of expansion media and media were replaced every 2 to 3 d. Basal media consisted of William E media containing 1% (vol/vol) glutamax, 1% (vol/vol) nonessential amino acids, 1% (vol/vol) penicillin/streptomycin (all from Gibco), 0.2% (vol/vol) normocin (Invivogen), 2% fetal bovine serum (FBS) (Omega), 2% (vol/vol) B27 (Gibco), 1% (vol/vol) N2 supplement (Gibco), 100 mM nicotinamide (Sigma-Aldrich), 1.25 mM *N*-acetylcysteine (Sigma-Aldrich), 10 μM Y27632 (Peprotech), and 1 μM A83-01 (Tocris). Expansion media contained 3 μM CHIR99021 (Peprotech), 25 ng/mL Epidermal Growth Factor (EGF) (Peprotech), 50 ng/mL Hepatocyte Growth Factor (HGF) (Peprotech), and 100 ng/mL Tumor Necrosis Factor alpha (TNFα) (Peprotech). To passage, cells were incubated with TrypLE Express (Gibco) for 5 min at 37 °C. Dissociated hepatocytes were transferred into new plates with fresh expansion media. Remaining cells were transferred to basal media and centrifuged at 300 × *g* for 4 min. Cells were resuspended in Bambanker (Wako) and stored at −80 °C for be thawed following standard procedures for subsequent cultures.

### Lentiviral Gene Delivery to Primary Hepatocytes.

At 24 h prior to transfection, HEK293T cells were plated in six-well plates at 8 × 105 cells per well in Dulbecco's Modified Eagle Medium (DMEM) (10% vol/vol FBS). Cells growing at ∼70 to 80% confluency were transfected with 1 μg of pLKO.1-puro-CMV-TurboGFP (Sigma-Aldrich) or one of five mouse *Tbx3* MISSION shRNAs (TRCN0000348157, TRCN0000348092, TRCN0000095870, TRCN0000334009, and TRCN0000095872) along with second generation lentiviral packaging and envelope plasmids, 0.75 μg of psPAX2 and 0.25 μg of pMDL.g (Addgene #12260, #12259, gifts from Didier Trono [Ecole Polytechnique Fédérale de Lausanne , 1015 Lausanne, Switzerland] ). At 36 h posttransfection, the media containing lentiviral particles were collected and passed through a 0.45-μm filter. Polybrene was added to a final concentration of 4 μg/mL. The filtered media containing lentivirus were added at a 1:1 (vol/vol) dilution to target primary hepatocytes. A second infection was performed at 60 h posttransfection. Hepatocytes were put on expansion media with puromycin (10 μL/mL) 1 d after the second transfection for 2 d for selection. Hepatocytes were harvested 4 d after the second transfection for qRT-PCR analysis and two out of five *Tbx3* shRNA were verified to be effectively knocking down *Tbx3*. A total of 50,000 cells from these two lines were plated for growth curve analysis. Cell counts were performed using the Cellometer K2 (Nexelcom).

### Generation of Tbx3-HA Ectopic Expression Vector.

*Tbx3* was amplified from the Mammalian Gene Collection (MGC) fully sequenced mouse *Tbx3* cDNA (Ge Dharmacon MMM1013-202797681) with forward primer: 5′-TAAGCTTCTGCAGGTCGACTATGAGCCTCTCCATGAGAGATCC-3′ and reverse primer: 5′-GAACATCGTATGGGTACATTGGGGACCCGCTGCAAGAC-3′ and inserted into pKH3(Addgene 12555) to add HA-tag at C-terminal of *Tbx3* by NEBuilder HiFi DNA Assembly Cloning Kit (E5520S). Then Tbx3-HA was amplified with forward primer: 5′-TGGAGGAGAACCCTGGACCTATGAGCCTCTCCATGAGAG-3′ and reverse primer: TGATCAGCGGGTTTAAACTCAGCGTAATCTGGAACATC-3′ and inserted into *pT2-SVNeo-EF1α-eGFP-P2A_PmeI* (modified from *pT2-SVNeo-EF1α-eGFP-P2A_EcoRI*, gift from Eric Rulifson [Stanford University, Stanford, CA]).

### Applying Sleeping Beauty Transposon System in Primary Hepatocytes.

Primary hepatocytes were cultured with expansion media till 60% confluent. The Sleeping Beauty System was applied as described in the plasmid DNA transfection protocol from TransIT-X2 Dynamic Delivery System (Mirus Biotech) with modifications. A total of 2.25 μg of *pT2-SVNeo-EF1α-eGFP* or *pT2-SVNeo-EF1α-eGFP-P2A-Tbx3-HA* along with the transposase in a ratio of 10:1 were mixed with *Trans*IT-X2 and incubated for 48 h. Then cells were put on G418 selection with expansion media for 48 h. Cells expressing *GFP* only or *GFP* together with *Tbx3* were expanded for growth curve or flow cytometry analysis.

### Hepatocyte Nuclei Isolation and Ploidy Analysis.

Hepatocyte nuclei preparation method was developed by modifying the chromatin preparation protocols described previously (63, 64). Liver lobes were homogenized in cold 1% formaldehyde in PBS with a loose pestle and dounce homogenizer with 15 to 20 strokes and fixed for 10 min at room temperature. Samples were incubated for 5 min with glycine at a final concentration 0.125 M and centrifuged at 300 × *g* for 10 min, at 4 °C. Pellets were washed in PBS and resuspended with 10 mL cell lysis buffer (10 mM Tris⋅HCl, 10 mM NaCl, 0.5% IGEPAL) and filtered through 100-μm cell strainers. A second round of homogenization was performed by 15 to 20 strokes with a tight pestle. Nuclei were pelleted at 2,000 × *g* for 10 min at 4 °C and resuspended in 0.5 mL PBS and 4.5 mL of prechilled 70% ethanol and stored at −20 °C for ploidy analysis. Nuclei were resuspended in 5 mL of PBS and approximately 1 million nuclei were stained with 0.5 mL of FxCycle PI/RNase (Thermo Fisher, F10797) staining solution for 15 to 30 min at room temperature. Primary hepatocytes were fixed and stained the same way. Both nuclei and cells were analyzed on FACS ARIA II (BD). Data were processed with FACS Diva 8.0 software (BD) and FlowJo v10 (FlowJo). Doublets were excluded by forward-scatter width (FSC-W)/ forward-scatter height (FSC-H) and side-scatter width (SSC-W) / side-scatter height (SSC-H) analysis. Single-stained channels were used for compensation and fluorophore minus one control was used for gating.

### ChIP-qPCR and Sequencing.

Three to five million *Tbx3* overexpressing primary hepatocytes were used for each ChIP reaction as recommended (65). Chromatin was prepared with a truChIP Chromatin Shearing Reagent Kit (Covaris 520154) and sheared with Covaris S220 according to the kit manual. A total of 4 μg anti-HA antibody (Roche Sigma 11867423001) was used for immunoprecipitation with a BioVision immunoprecipitation kit (K286-25). Rat IgG1 isotype control (Thermo Fisher MA1-90035) was used for IgG control. ChIP washing steps, input, and ChIP DNA preparation were modified as described previously (64) and sent for sequencing through the NextSeq 500 system (Illumina). Sequencing data were mapped as previously described with modifications (65). Raw reads were mapped with Bowtie 2 (66) and processed and sorted with Samtools (67). Peaks were called using MACS2 as previously described (68–70) with modifications from subcommands. Purified input, IgG, and ChIP DNA following the chromatin immunoprecipitation were also used in ChIP-qPCR and calculated as described previously (63). Forward: 5′-AAGGTCATCCCAGAGCTGAA-3′ and reverse: 5′-CTGCTTCACCACCTTCTTGA-3′ primers detecting *Gapdh* promoter area were used as internal controls. *E2f7* forward: 5′-CGCCAGAGGAGGTGTTTTAG-3′ and reverse: 5′-CGGGCAGAGCGTGTAGAT-3′ or *E2f8* forward: 5′-GAACACTTGGGTGACCCTGA-3′ and reverse: 5′-CAAAGGGAATGCACACTGG-3′ were used to detect the TBX3-bound *E2f7* or *E2f8* promoter areas, respectively.

### Luciferase Assay for Promoter Function.

The HSV-TK promoter was cloned upstream of luciferase in the pGL4.10[luc2] plasmid (Promega). A 122-bp region of the *E2f7* promoter or a 2.1-kb region of the *E2f8* enhancer was cloned into the *Xho*I site upstream of the HSV-TK promoter. Two T-box binding motifs from the *E2f7* promoter were mutated and six motifs were deleted from the *E2f8* enhancer. HepG2 cells were cotransfected with 250 ng of the test plasmid and 250 ng of control pRL-TK plasmid, using the TransIT-X system (Mirus). A dual luciferase reporter assay (Promega) was performed at 48 h posttransfection with luminescence detected by a luminometer (Berthold Technologies). Firefly luciferase activity was normalized to Renilla luciferase in each sample.

### Quantification of Nuclear Area.

Liver tissues were sectioned at 5 μm, stained with anti-GFP or anti-HNF4α antibodies, and imaged at 20× magnification. Hepatocyte nuclei were detected automatically with the “analyze particles” feature of Fiji (ImageJ) software to encircle GFP- or HNF4α-positive nuclei and selected regions were validated or corrected manually when necessary. Area of each encircled nucleus was then measured with the “measure” feature. The number of nuclei measured per animal (*n* = 3 mice, unless otherwise noted) was as follows: control = 267 to 339 and Tbx3 KO = 222 to 310 (Fig. 2*C*); control week 2 *=* 360 to 392 and Tbx3 KO week 2 *=* 341 to 431; control week 3 *=* 436 to 714 and Tbx3 KO week 3 *=* 298 to 714; control weeks 9 to 12 *=* 278 to 411 and Tbx3 KO weeks 9 to 12 *=* 182 to 399 (Fig. 2*G*); control *=* 265 to 338 and Tbx3-E2f7-E2f8 TKO *=* 303 to 370, *n* = 4 mice (Fig. 4*D*); control *=* 348 to 375, Tbx3 KO *=* 303 to 409 and Tbx3-E2f7-E2f8 TKO *=* 493 to 626 (*SI Appendix*, Fig. S4*C*).

### Fibrosis Assay.

Livers were fixed in 10% neutral buffered formalin and sectioned at 5-μm thickness. Slides were deparaffinated in HistoClear (Natural Diagnostics), hydrated in graded ethanol series, and rinsed in distilled water. Slides were covered in 0.1% Sirius Red in saturated picric acid F-357-2 (Rowley Biochemical) for 1 h and washed in two changes of acidified water (0.5% glacial acetic acid). They were then dehydrated in ethanol, cleared in HistoClear, and mounted in resinous medium. Brightfield images were collected on a Zeiss Imager Z.2.

## Supplementary Material

Supplementary File

## Data Availability

All study data are included in the article and/or supporting information. The TBX3 ChIP-Sequencing dataset is available in the Gene Expression Omnibus (GEO) with the accession number GSE205339 (71) or the link https://www.ncbi.nlm.nih.gov/geo/query/acc.cgi?acc=GSE205339.
