# Supplementary material for: Wnt signaling regulates hepatocyte cell division by a transcriptional repressor cascade
Source: Proc Natl Acad Sci U S A. 2022 Jul 22;119(30):e2203849119. doi: 10.1073/pnas.2203849119 (PMC9335208; doi:10.1073/pnas.2203849119)
Supplement: Supplementary File [file pnas.2203849119.sapp.pdf]

Supplementary Information for

Wnt Signaling Regulates Hepatocyte Cell Division by a Transcriptional Repressor Cascade

Yinhua Jin[a,b,1], Teni Anbarchian[a,b,1], Peng Wu[a,b,c] , Abby Sarkar[a,b], Matt Fish[a,b], Weng Chuan Peng[a,b,2], Roel Nusse[a,b,3]

[a]Department of Developmental Biology, Institute for Stem Cell Biology and Regenerative Medicine, Stanford University School of Medicine, Stanford, CA 94305;
[b]HHMI, Stanford University School of Medicine, Stanford, CA 94305; and
[c]Department of Pediatrics, Stanford University School of Medicine, Stanford, CA 94305

[1]Y.J. and T.A. contributed equally to this work.
[2] Present address: Princess Máxima Center for Pediatric Oncology, 3584 CS, Utrecht, The Netherlands.
[3]To whom correspondence may be addressed: Email: rnusse@stanford.edu

This PDF file includes:

Supplementary text
Supplementary Figures 1 to 4
Supplementary Table 1

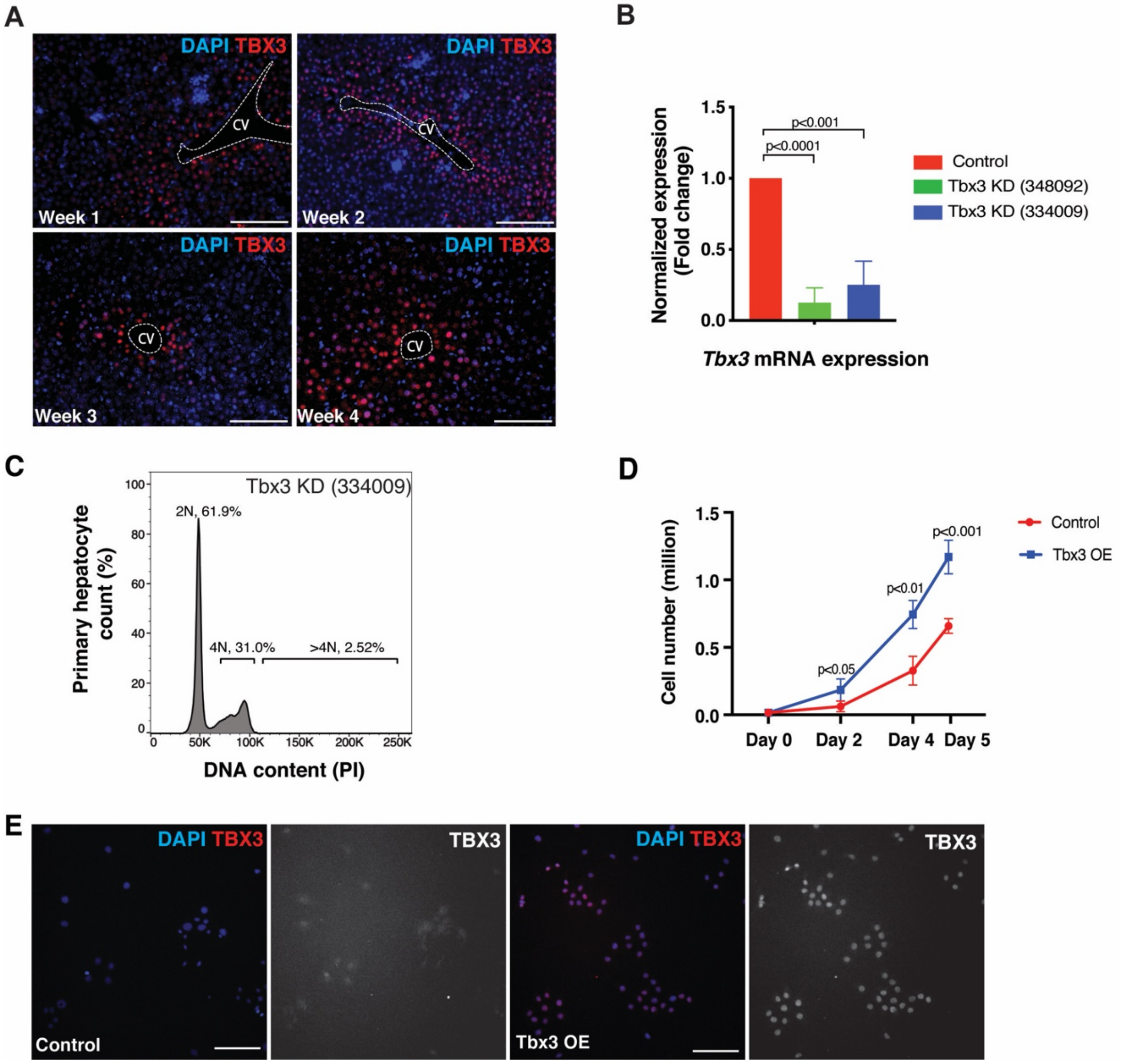

Figure S1. Expression of *Tbx3 in vivo* and its modulation *in vitro.* (A) *Tbx3* is expressed broadly in the pericentral zone during the postnatal growth phase. TBX3 protein is detected by immunofluorescence in hepatocytes surrounding the central veins, at postnatal weeks 1-4. Representative images are shown. (B) Efficient knockdown (KD) of *Tbx3* in hepatocytes. Cultured mouse primary hepatocytes were treated with control or one of two different Tbx3 shRNA constructs (# #348092 and #334009). Normalized fold change in expression of *Tbx3* mRNA levels measured by qRT-PCR is shown. Statistical significance was determined by Student's t-test between control and each Tbx3 KD condition from n=3 independent experiments. (C) Tbx3 KD with shRNA #334009 resulted in a minor increase in the percentage of polyploid cells, compared to Control in Fig. 1C. Representative flow cytometry plots show ploidy distribution of Tbx3 KD cells, stained with propidium iodide (PI). Percentages of ploidy classes are reported as averages from n=3 independent experiments. (D) *Tbx3* overexpression (OE) leads to increased hepatocyte proliferation in culture. Growth curves of Control *(EF1α-GFP)* and Tbx3 OE (*EF1α-GFP-P2A-Tbx3*) primary hepatocytes are shown at days 0, 2, 4 and 5 after seeding. Statistical significance was determined by Student's t-test from n=3 independent experiments. (E) Cultured Tbx3 OE primary hepatocytes. Representative Tbx3 immunofluorescence images of Control or Tbx3 OE mouse primary hepatocytes are shown. Scale bars, 100μm. CV, central vein. KD, knock down. OE, overexpression. PI, propidium iodide. Dashed lines delineate central veins.

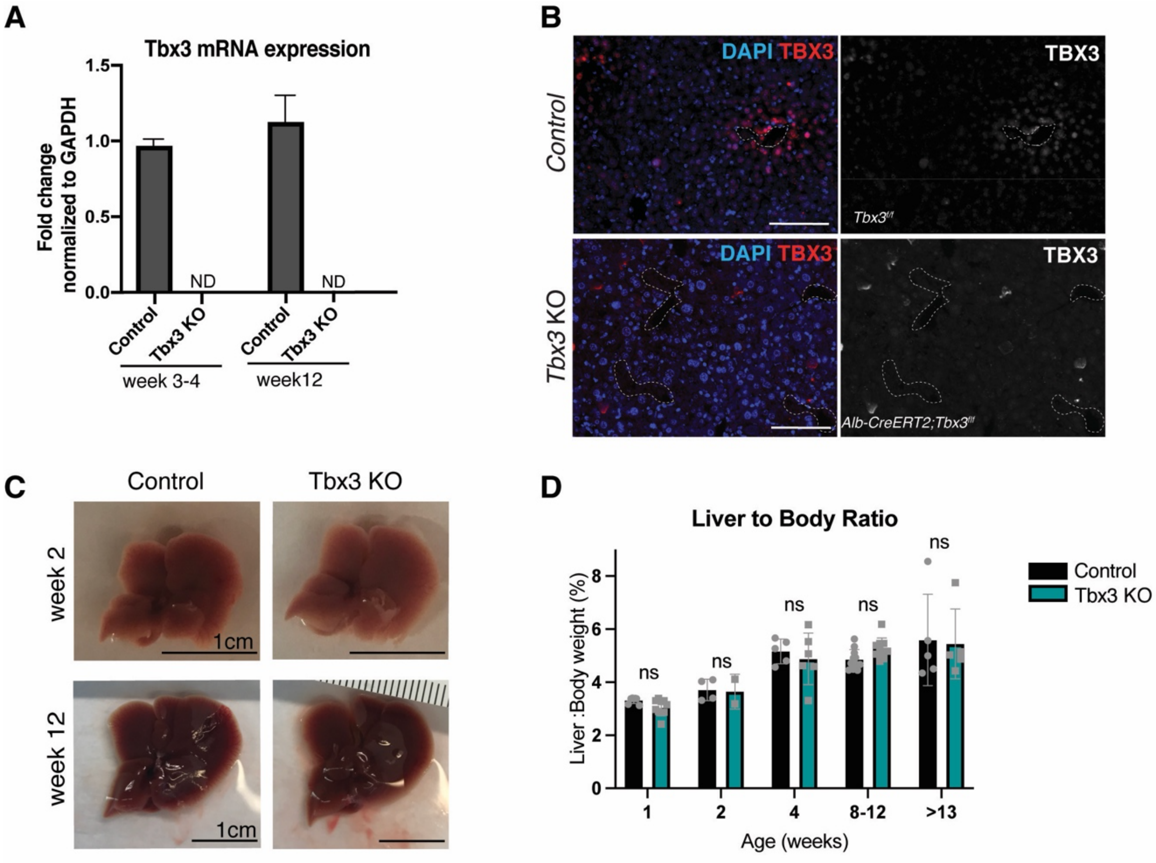

Figure S2. AlbCreERT2-driven loss of *Tbx3* does not impact liver size or morphology. (A) Efficient deletion of Tbx3 mRNA with the Alb-CreERT2 driver. Neonates received a single dose tamoxifen at postnatal day 3 and Tbx3 deletion was verified by qRT-PCR in Control *($Tbx3^{f/f}$)* and Tbx3 KO *(Alb-CreERT2; $Tbx3^{f/f}$)* livers at postnatal weeks 3-4 and 12. (B) Efficient deletion of Tbx3 protein with the Alb-CreERT2 driver. Representative images of Tbx3 immunofluorescence, at postnatal week 3 in Control *($Tbx3^{f/f}$)* and Tbx3 KO *(Alb-CreERT2; $Tbx3^{f/f}$)* livers are shown. (C) Photographs of Control *($Tbx3^{f/f}$)* and Tbx3 KO *(Alb-CreERT2; $Tbx3^{f/f}$)* livers at postnatal weeks 2 and 12 show no changes to liver morphology. (D) Liver to body weight ratios at various weeks after birth show no significant differences in liver-to-body weight ratio in between Control *($Tbx3^{f/f}$)* and Tbx3 KO *(Alb-CreERT2; $Tbx3^{f/f}$)* animals. Each data point is representative of one animal. ND, Not Detected. ns= not significant. Dashed lines delineate veins.

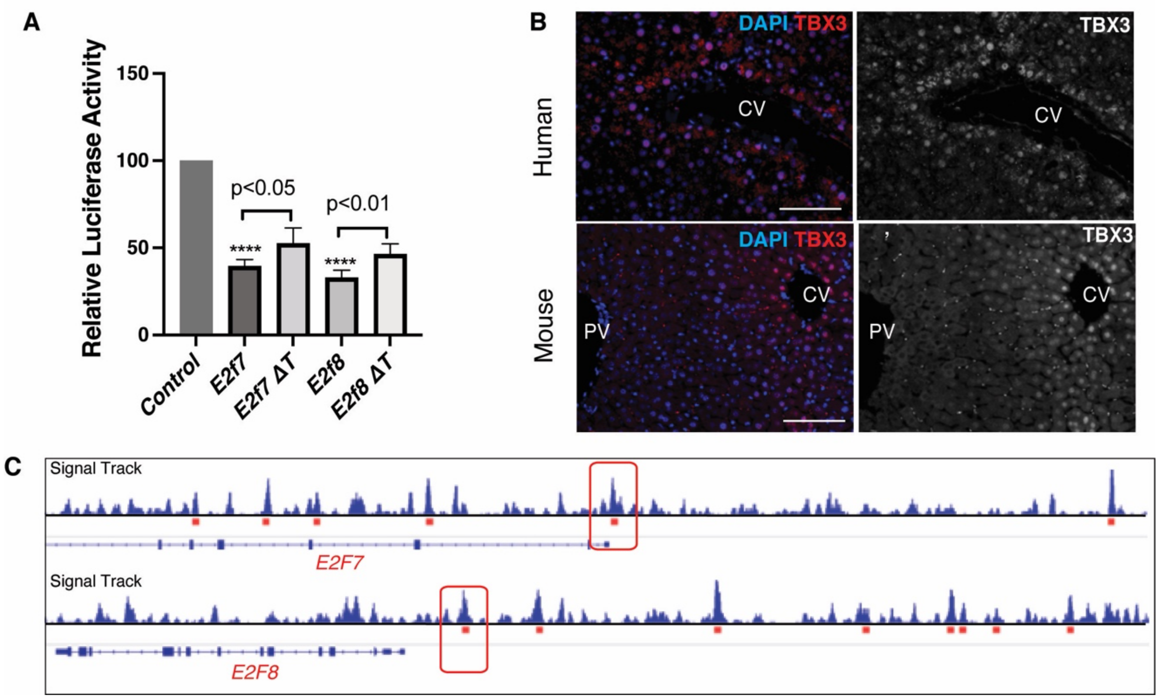

Figure S3. TBX3 as a specific repressor of E2F7/E2F8, is expressed in pericentral hepatocytes of the human liver and binds to E2F7/E2F8 in human hepatocytes. (A) Tbx3 represses E2F7 and E2F8 by binding to T-box motifs. T-box motifs were mutated on E2F7 and deleted on E2F8 loci and were cloned into luciferase expression constructs for functional analyses in HepG2 cells and luciferase activity was measured. P-values noted via asterisks are comparisons of Control vs. E2F7 and Control vs. E2F8 in the Vector control condition. ****P<0.0001. Statistical significance was determined by Student's t-test from n=5 independent experiments. Error bars indicate standard deviation. (B) Tbx3 is expressed in the pericentral hepatocytes of human livers. TBX3 protein is detected by immunofluorescence in both human and mouse livers. (C) Tbx3 binds to E2F7 and E2F8 in human hepatocytes. Tbx3 Chromatin Immunoprecipitation-Sequencing (ChIP-Seq) Signal Track from HepG2 Cells (ENCODE) for E2F7 and E2F8 loci. Regions underlined in red indicate significant binding peaks. Boxed regions are significant binding regions conserved in mouse. CV, Central Vein. PV, Portal Vein. Scale bars, 100 μm.

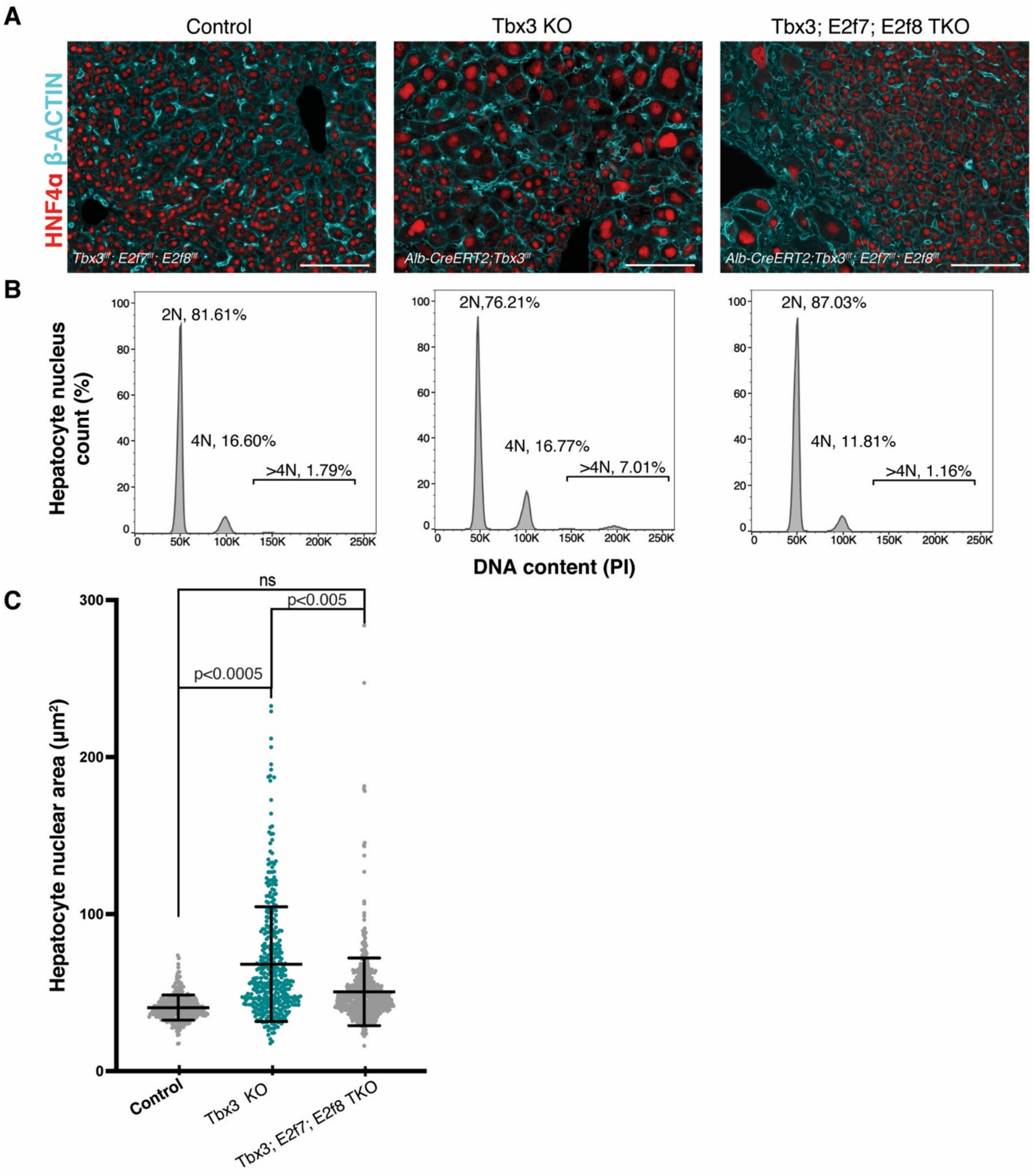

Figure S4. Genetic epistasis test showing Tbx3 controls hepatocyte polyploidy by repressing *E2f7* and *E2f8*. (A) Membrane and nuclear visualization of Control or Alb-CreERT2-driven Tbx3 KO or Tbx3-E2f7-E2f8 TKO livers. Representative Hnf4α and β-Actin immunofluorescence images at postnatal week 4 are shown. (B-C) Deletion of *E2f7* and *E2f8* along with *Tbx3* rescues the polyploidy phenotype of Tbx3 KO. (B) Representative flow cytometry plots of nuclear ploidy distribution from n=5 Control *(Tbx3$^{f/f}$; E2f7$^{f/f}$; E2f8$^{f/f}$)*, n=4 Tbx3 KO *(Alb-CreERT2; Tbx3$^{f/f}$)* and n=4 Tbx3-E2f7-E2f8 TKO *(Alb-CreERT2; Tbx3$^{f/f}$; E2f7$^{f/f}$; E2f8$^{f/f}$)* livers at postnatal week 4. (C) Measurements of hepatocyte nuclear area in livers from (A). n=3 mice pre genotype. Bars indicate mean and standard deviation for all measured nuclei per genotype. Means of nuclear area were calculated for each animal. Means for each genotype were compared by one-way ANOVA. Ns= not significant (adjusted P=0.0626). Scale bars, 100μm. PI, propidium iodide.

Table S1. Average ploidy distribution of hepatocyte nuclei.

Genotype	Average			p-Value		
	2N%	4N%	>4N%	2N%	4N%	>4N%
Control (GFP)	76.7	19.5	1.4	$p < 0.001$	$p < 0.05$	$p < 0.05$
Tbx3 KD (shRNA #348092)	57.1	32.4	5.7			
Control (EF1α-GFP)	42.1	50.6	5.4	$p < 0.001$	$p < 0.001$	$p < 0.05$
Tbx3 overexpressing (Tbx3 OE, EF1α-GFP-P2A-Tbx3)	67.4	27.3	2.5			
Axin2-rtTA; TetO-H2B-GFP; Tbx3$^{f/f}$	39.3	48.6	11.2	$p < 0.001$	$p < 0.05$	$p < 0.005$
Axin2-rtTA; TetO-H2B-GFP; TetO-Cre; Tbx3$^{f/f}$	7.6	63	29			
Axin2-rtTA; TetO-H2B-GFP; Tbx3$^{f/f}$; E2f7$^{f/f}$; E2f8$^{f/f}$	43.0	55.7	1.3	ns	ns	ns
Axin2-rtTA; TetO-H2B-GFP; TetO-Cre; Tbx3$^{f/f}$; E2f7$^{f/f}$; E2f8$^{f/f}$	53.4	45.4	1.1			
Tbx3$^{f/f}$	84.3	12.3	1.0	$p < 0.05$	$p < 0.05$	ns
Alb-CreERT2; Tbx3$^{f/f}$	52.3	39.2	7.3			
Tbx3$^{f/f}$; E2f7$^{f/f}$; E2f8$^{f/f}$	81.6	16.6	1.8	ns	ns	ns
Alb-CreERT2; Tbx3$^{f/f}$; E2f7$^{f/f}$; E2f8$^{f/f}$	87.0	11.9	1.2			
